# Multimodal cardiovascular model for hemodynamic analysis: Simulation study on mitral valve disorders

**DOI:** 10.1371/journal.pone.0247921

**Published:** 2021-03-04

**Authors:** Dibyendu Roy, Oishee Mazumder, Aniruddha Sinha, Sundeep Khandelwal

**Affiliations:** TCS Research, Tata Consultancy Services Limited, Kolkata, India; Scuola Superiore Sant’Anna, ITALY

## Abstract

Valvular heart diseases are a prevalent cause of cardiovascular morbidity and mortality worldwide, affecting a wide spectrum of the population. In-silico modeling of the cardiovascular system has recently gained recognition as a useful tool in cardiovascular research and clinical applications. Here, we present an in-silico cardiac computational model to analyze the effect and severity of valvular disease on general hemodynamic parameters. We propose a multimodal and multiscale cardiovascular model to simulate and understand the progression of valvular disease associated with the mitral valve. The developed model integrates cardiac electrophysiology with hemodynamic modeling, thus giving a broader and holistic understanding of the effect of disease progression on various parameters like ejection fraction, cardiac output, blood pressure, etc., to assess the severity of mitral valve disorders, naming *Mitral Stenosis* and *Mitral Regurgitation*. The model mimics an adult cardiovascular system, comprising a four-chambered heart with systemic, pulmonic circulation. The simulation of the model output comprises regulated pressure, volume, and flow for each heart chamber, valve dynamics, and Photoplethysmogram signal for normal physiological as well as pathological conditions due to mitral valve disorders. The generated physiological parameters are in agreement with published data. Additionally, we have related the simulated left atrium and ventricle dimensions, with the enlargement and hypertrophy in the cardiac chambers of patients with mitral valve disorders, using their Electrocardiogram available in Physionet PTBI dataset. The model also helps to create ‘what if’ scenarios and relevant analysis to study the effect in different hemodynamic parameters for stress or exercise like conditions.

## Introduction

Cardiovascular diseases (CVD) account for a massive rate of mortality all over the world. Recent statistics from World Health Organization (WHO) reported nearly 17.9 million death due to cardiovascular disease along with the economic burden of billions of dollars spent every year on screening, diagnosis, and related health-care-associated to CVD [1]. Out of various CVDs, valvular heart disease (VHD) on the left ventricle is a prominent cause of cardiovascular morbidity and mortality worldwide [2]. The aging population is mostly affected by degenerative valve disease, whereas in developing countries, rheumatic valve disease remains a public health problem affecting young adults [3].

Out of the valvular disease associated with the left ventricle, dysfunction of the mitral valve remains a leading medical problem worldwide [4]. This valve plays a fundamental role in the structural and functional integrity of the left ventricle, along with maintaining forward cardiac output. Dysfunction may arise due to structural defects in any of the valve structures, causing ‘stenosis’ or ‘regurgitation’ resulting in symptoms like ventricular hypertrophy, atrial enlargement, reduced cardiac output, pulmonary venous congestion, and atrial arrhythmia [5]. Computational modeling may provide an intuitive platform to understand the biomechanics of the human mitral valve along with changes in hemodynamic parameters with progression of the disease that could eventually lead to the development of new treatment, prevention, and diagnosis of mitral valve disease.

Recently, cardiovascular research has shown significant involvement in simulating cardiac behavior through in-silico models [6]. Powered by improvement in computing technologies, cardiac modeling based on physical principles are being used to simulate the hemodynamic properties of the cardiovascular system [7]. These models are imparting an increasingly important role in the diagnosis of cardiovascular diseases along with the development of medical devices [8]. Although substantial research exists on modeling mechanisms related to hemodynamics, electrophysiology, computational fluid dynamics, biomechanics, etc., researchers are now focusing on the multiscale mathematical framework to simulate the cardiac function at the whole-organ scale [9]. In the hemodynamics domain, many analytical representations of the cardiovascular system have been proposed since the first system-level dynamic cardiovascular model [10]. Depending on the purpose of the underlying scientific questions, hemodynamic analysis vary from simple lumped models, 0-1D multiscale cardiovascular model to complex 3D image-based models [11]. Some of the recent models are the fluid-structure interaction in specific vascular beds [12], the distributed impedance of the arterial and pulmonary trees [13], and lumped models of the integrated cardiovascular system [14]. A particular area in hemodynamics that has received substantial attention is one-dimensional reduced-order models. These models are commonly used to simulate blood flow regimes for which pulse waves propagate in large compliant arteries [15]. These models are computationally efficient and mostly linked to study the physiological hemodynamic phenomena under various pathological conditions [16, 17]. In the integrated multimodal modeling domain, there are some hemodynamic models based on cellular properties such as ion equations, myofilament structure, and fiber orientation [18]. However, computational load inhibits these models from use in clinical and educational applications of central hemodynamics [19]. Computational models related to the mitral valve mostly concentrate on the structural variation of the valve with fluid-structure interaction [20]. Such models might aid in surgical intervention but lack the holistic integration of pressure and flow dynamics in the heart chamber, caused due to structural defects of the valve [21]. Models focusing on mitral valve dynamics and heart remodeling [22] offer good examples of how cardiovascular simulation models can be validated in specific situations and used clinically.

**Fig 1:**
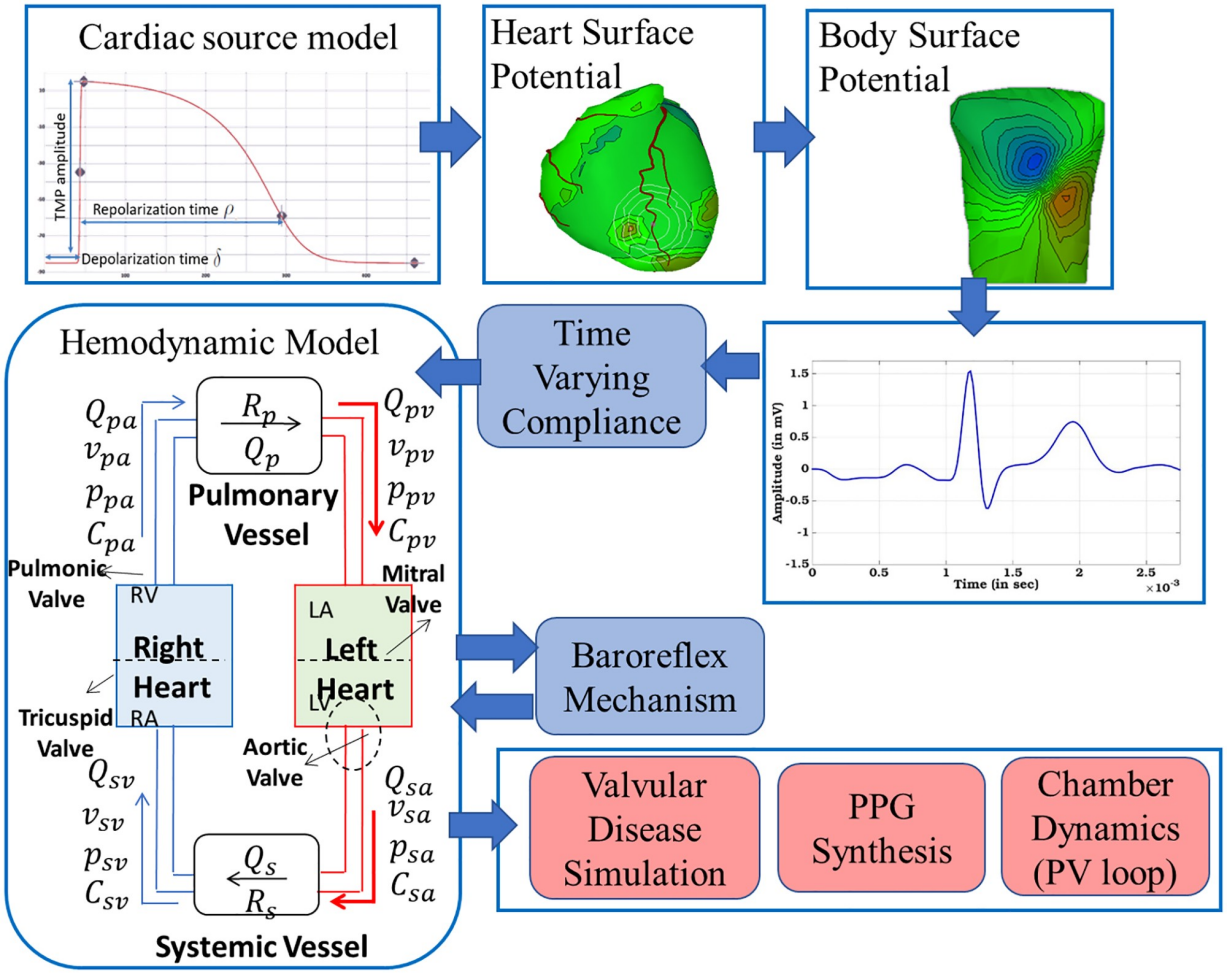
Schematic diagram of the developed electrophysiology driven cardiovascular hemodynamic model.

In this paper, we present a closed-loop, real-time, lumped parameter cardiovascular simulation model, which contains the dynamics of four cardiac chambers, heart valves, and lumped pulmonic and systemic circulation. It is a reduced order model, where the pulsatile behavior of the heart chambers is triggered and modulated through cardiac electrophysiology. The forward electrophysiology pipeline (EP) has been implemented to generate a single lead electrocardiograph (ECG) signal from a cardiac source model. Hemodynamics is governed through time-varying compliance as derived from the EP model. A schematic workflow of the developed prototype is shown in Fig 1. We hypothesize that the working of the cardiac hemodynamics can be approximated by a reduced-order lumped model using pressure-flow variation at different instances of the cardiac cycle. We aim to replicate the normal cardiac hemodynamics physiology and then incorporate pathological conditions pertaining to mitral valve disorders and correlate the parametric variation of hemodynamic indices simulated by our model against values reported in the medical literature.

Cardiac dynamics incorporate both active and passive filling phases resulting in more realistic changes of pressures with ventricular dilatation. Valve dynamics and precise adjustment of valvular properties allow realistic simulation of both stenotic and insufficient valves. The hemodynamics is coupled with central nervous system through sympathetic and parasympathetic control via baroreflex auto-regulation mechanism. The integrated EP-hemodynamics approach takes into account the cellular to organ level manifestation of cardiac dynamics, thus making the model multimodal and multiscale. Such prototype model can be used to simulate the cardiac dynamics for healthy physical condition as well as mitral valve disorders, namely ‘stenosis’ and ‘regurgitation’ with varying severity. The model is used to simulate the pressure-volume (PV) dynamics of the left ventricle and atrium. This in turn provides the information on the stroke volume, variations in aortic and pulmonary pressure and indications of hypertrophy/enlargement in cardiac chambers. Additionally, as part of modeling the systemic circulation, variations in the peripheral blood volume pulse are used to simulate the Photoplethysmogram (PPG) signals [23]. Such PPG signal, in turn, could be used to correlate with observed PPG signals of an individual, and hence be used for early screening applications for CVD [24].

We demonstrate that the proposed model generates cardiac parameters, for both normal and pathophysiological cases, which are consistent with earlier published clinical and experimental data. Additionally, the model has been tested using a limited set of measured ECGs of patients with mitral valve disorders. In such a scenario, the measured ECG serves as an input to the model to generate hemodynamic parameters. A subset of these parameters namely, the dimensions of the left cardiac chambers are validated with the left ventricular hypertrophy and left atrial enlargement score, obtained from such clinical data.

The rest of the paper is organized as follows. Section 2 comprises the methodology, which includes the layout of the cardiovascular hemodynamic model containing the four-chambered heart, followed by the EP model to generate ECG signal from a cardiac source model and subsequently deriving the chamber and valve dynamics, and PPG synthesis for a healthy adult. In section 3, we simulate the mitral valve stenosis and regurgitation of varying severity followed by section 4, where we present the simulation results for healthy, mitral stenosis and regurgitation condition and a hypertrophy or enlargement analysis based on real ECG data. In section 5, we discuss the capability of ‘what if’ simulation through a simulated stress condition and report its effect on mild mitral valve disorders followed by a brief discussion on the capability and limitations of our model. Section 6 concludes the paper.

## Methodology

The developed cardiovascular model integrates two separate modalities, namely the hemodynamic model and a reduced electrophysiological model, sufficient to derive the compliance function for driving the hemodynamic model. In this section, the layouts of the cardiovascular hemodynamic model of the four-chambered heart along with the EP model, and PPG synthesis have been discussed.

### Mathematical model of cardiovascular hemodynamic system

**Fig 2:**
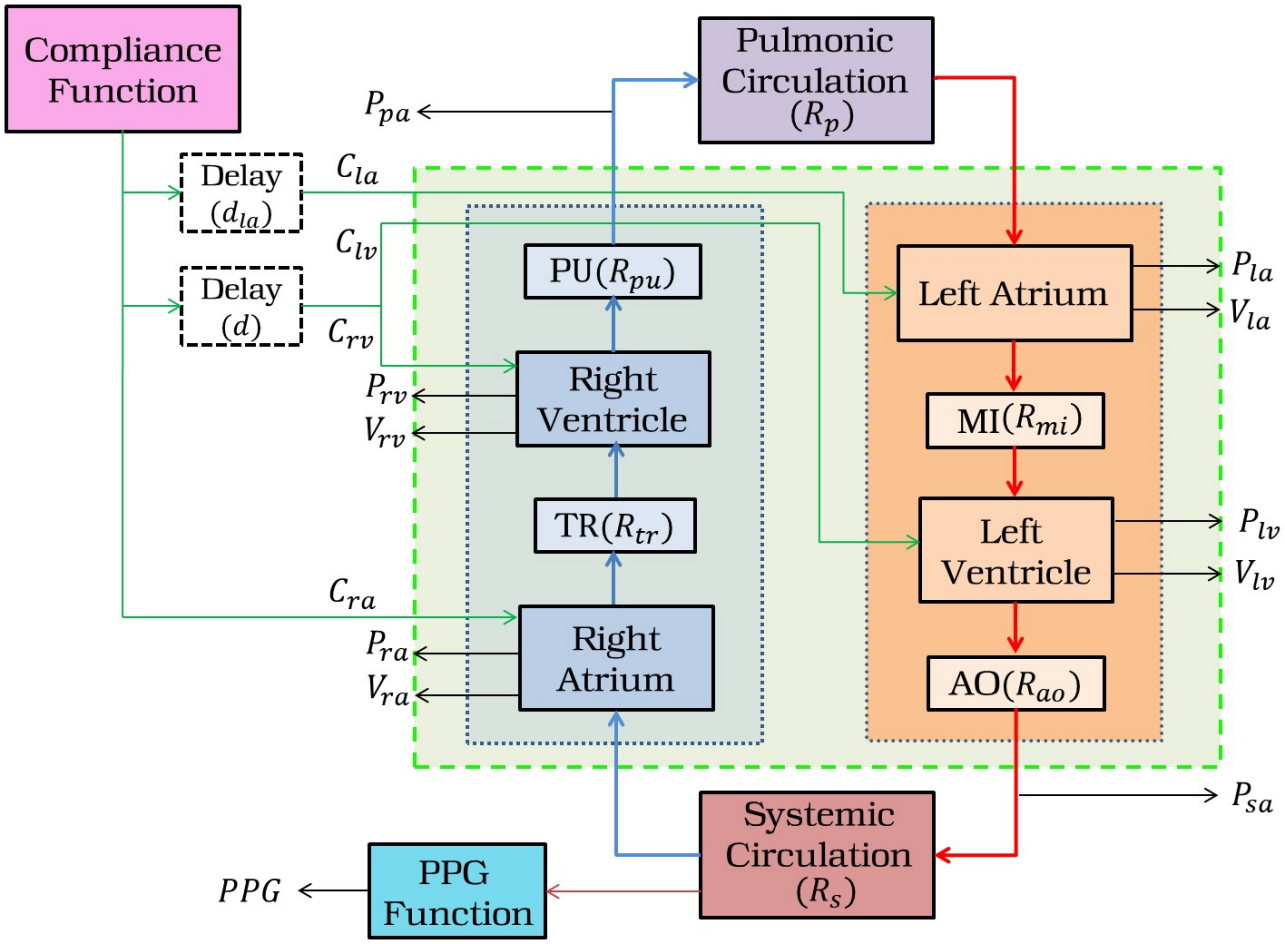
Block diagram of the cardiovascular system. *P*_*la*_, *V*_*la*_, *P*_*lv*_, *V*_*lv*_, *P*_*ra*_, *V*_*ra*_, *P*_*rv*_, *V*_*rv*_ are the pressure and volume in the left atrium and ventricle, right atrium and ventricle respectively. *P*_*sa*_ and *P*_*pa*_ are the pressures in the systemic and pulmonary artery respectively. *C*_*ra*_, *C*_*la*_, *C*_*lv*_, and *C*_*rv*_ are the compliances across right atrium, left atrium, left ventricle, and right ventricle respectively, with associated delays of *d*_*la*_ and *d*. The resistances across pulmonic and systemic vessels are *R*_*p*_, *R*_*s*_ respectively. *R*_*mi*_, *R*_*ao*_, *R*_*tr*_, and *R*_*pu*_ are the valvular resistances for the mitral (MI), aortic (AO), tricuspid (TR) and pulmonary (PU) valves respectively.

The heart is a muscular organ in which each half is composed of a pair of atrium and ventricle acting like a pulsatile pump. The left heart chamber, comprising the left ventricle (*lv*) and left atrium (*la*), pumps oxygenated blood to all the tissues of the body. This specific circulation is called systemic circulation [25]. On the other hand, the right heart, comprising right ventricle (*rv*) and right atrium (*ra*), drives deoxygenated blood to the lungs forming the pulmonic circulation. In addition, there are four cardiac valves namely, mitral (*mi*), aortic (*ao*) valves in the left heart and tricuspid (*tr*), pulmonic (*pu*) valves in the right heart respectively. These valves synchronously open and close based on the pressure difference within the heart chambers and ensures rhythmic unidirectional flow through the heart. The block-diagrammatic representation is shown in Fig 2.

To describe the hemodynamics of the cardiac system, we have considered the following assumptions [25]:

**Assumption 1**: Each of the heart chambers is triggered by an autonomous compliance function (*C*(*t*)), due to the elasticity of the cardiac walls. So, the volume (*V*(*t*)), at the time *t*, across any cardiac chamber can be defined as *V*(*t*) = *C*(*t*) × *P*(*t*) + *V*_*s*_; where *P*(*t*) is the pressure at the *t*^*th*^ time and *V*_*s*_ is the fixed unstressed volume of that particular chamber.
**Assumption 2**: The cardiac chambers are considered as compliance vessels. Hence, the rate of change of volume across a cardiac chamber at time *t*, can be defined as the difference between the inflow *Q*_1_(*t*) and the outflow *Q*_2_(*t*), so, $\frac{dV(t)}{dt}=Q_{1}\left(t\right)-Q_{2}\left(t\right)$.
**Assumption 3**: Each vessel is considered as resistive vessel, as the blood flow is impeded due to the frictional forces, depending on the viscosity of the blood, diameter of the vessels etc. Thus, flow across a resistive vessel will be *Q*(*t*) = Δ*P*(*t*)/*R*; where Δ*P*(*t*) is the pressure difference in the successive compartments of the vessel, at the time *t* and *R* is the vascular resistance of that vessel.

Based on these assumptions, the pressure dynamics, replicating the left-heart hemodynamics can analytically be defined as given by Eqs (1)–(3) [26]).

$$\begin{array}{c} \begin{array}{cc} {\dot{P}}_{la}= & \frac{1}{C_{la}\left(t\right)}[\frac{P_{la}-P_{pa}}{R_{p}}-U_{mi}\times \frac{P_{la}-P_{lv}}{R_{mi}}-{\dot{C}}_{la}\left(t\right)P_{la}] \end{array} \end{array}$$ (1)

$$\begin{array}{c} \begin{array}{cc} {\dot{P}}_{lv}= & \frac{1}{C_{lv}\left(t\right)}[U_{mi}\times \frac{P_{la}-P_{lv}}{R_{mi}}-U_{ao}\times \frac{P_{lv}-P_{sa}}{R_{ao}}-{\dot{C}}_{lv}\left(t\right)P_{lv}] \end{array} \end{array}$$ (2)

$$\begin{array}{cc} {\dot{P}}_{sa}= & \frac{1}{C_{sa}}[U_{ao}\times \frac{P_{lv}-P_{sa}}{R_{ao}}-\frac{P_{sa}-P_{ra}}{R_{s}}] \end{array}$$ (3)

Similarly, the pressure dynamics describing the right-heart functionality can be interpreted by Eqs (4)–(6).

$$\begin{array}{c} \begin{array}{cc} {\dot{P}}_{ra}= & \frac{1}{C_{ra}\left(t\right)}[\frac{P_{sa}-P_{ra}}{R_{s}}-U_{tr}\times \frac{P_{ra}-P_{rv}}{R_{tr}}-{\dot{C}}_{ra}\left(t\right)P_{ra}] \end{array} \end{array}$$ (4)

$$\begin{array}{c} \begin{array}{cc} {\dot{P}}_{rv}= & \frac{1}{C_{rv}\left(t\right)}[U_{tr}\times \frac{P_{ra}-P_{rv}}{R_{tr}}-U_{pu}\times \frac{P_{rv}-P_{pa}}{R_{pu}}-{\dot{C}}_{rv}\left(t\right)P_{rv}] \end{array} \end{array}$$ (5)

$$\begin{array}{cc} {\dot{P}}_{pa}= & \frac{1}{C_{pa}}[U_{pu}\times \frac{P_{rv}-P_{pa}}{R_{pu}}-\frac{P_{la}-P_{pa}}{R_{p}}] \end{array}$$ (6)

Here *P*_*la*_, *P*_*lv*_, *P*_*sa*_, *P*_*ra*_, *P*_*rv*_ and *P*_*pa*_ are the pressure variables in the *la*, *lv*, systemic arteries (*sa*), *ra*, *rv*, and pulmonary arteries (*pa*) respectively, having the initial conditions of $p_{la}^{0}$, $p_{lv}^{0}$, $p_{sa}^{0}$, $p_{ra}^{0}$, $p_{rv}^{0}$ and $p_{pa}^{0}$. The valvular resistance across the mitral, aortic, tricuspid, and pulmonic valves are *R*_*mi*_, *R*_*ao*_, *R*_*tr*_, and *R*_*pu*_ respectively. The vascular resistance and compliance pair, across the pulmonic and systemic vessels are *R*_*p*_, *C*_*pa*_ and *R*_*s*_, *C*_*sa*_ respectively. *U*_*mi*_, *U*_*ao*_, *U*_*tr*_, and *U*_*pu*_ are the control inputs for opening and closing of the heart valves. The functionalities of these valves are defined by Eqs (7) and (8), where 1 represents the complete opening of the cardiac valves and *δ*_*i*_;∀*i* ∈ {*mi*, *ao*, *tr*, *pu*} represents the closing of the same. In healthy cardiac condition, *δ*_*i*_ = 0. In such a scenario, whenever the left-atrium pressure (*P*_*la*_) is greater than the left-ventricle pressure (*P*_*lv*_), then the mitral valve opens and *U*_*mi*_ is considered as 1. Similar is the case for other valves.

$$\begin{array}{c} U_{mi}=\{\begin{array}{c} 1,\;\text{if,}\;P_{la}>P_{lv}\\ \delta_{mi},\;\text{otherwise} \end{array};\;U_{ao}=\{\begin{array}{c} 1,\;\text{if,}\;P_{lv}>P_{sa}\\ \delta_{ao},\;\text{otherwise} \end{array}; \end{array}$$ (7)

$$\begin{array}{c} U_{tr}=\{\begin{array}{c} 1,\;\text{if,}\;P_{ra}>P_{rv}\\ \delta_{tr},\;\text{otherwise} \end{array};\;U_{pu}=\{\begin{array}{c} 1,\;\text{if,}\;P_{rv}>P_{pa}\\ \delta_{pu},\;\text{otherwise} \end{array}; \end{array}$$ (8)

**Fig 3:**
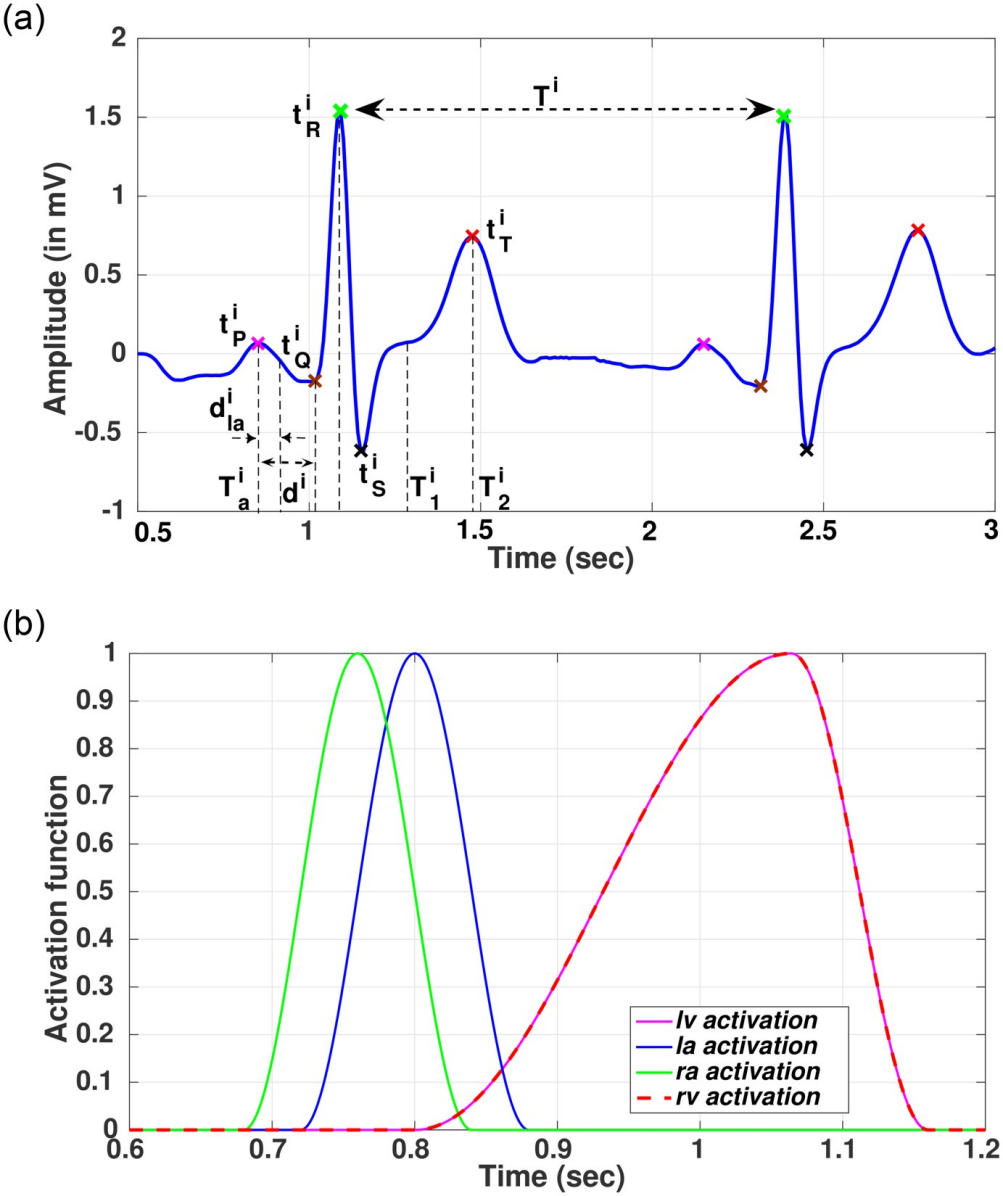
ECG simulation through EP forward pipeline and activation of cardiac chambers. (a) Simulated ECG signal for the *i*^*th*^ cardiac cycle. (b) Activation of cardiac chambers from ECG signal.

As per the *Assumption* 1, the heart chambers are activated sequentially, in a synchronized manner, by time-varying compliance functions. Typically, this activation starts from sinoatrial node [27], which is located inside *ra*, then, it traverses to the *la* with a time delay of *d*_*la*_, causing them to contract for pumping the blood into the ventricles. After that, the activation traverses from the atrium to the ventricles via atrioventricular node [27] with a time delay of *d* (Fig 3a), allowing the ventricles to fill with blood. To replicate these phenomena, we map the output of the EP source model, described later, to the compliance of the cardiac chambers, using a non-linear function. Among many options for such a mapping function [28], we have considered a cosine function which is capable of modeling the activation of cardiac chambers in both diseased and healthy heart [29]. Using such a mapping function, we have defined the compliance functions as *C*_*ra*_(*t*), *C*_*la*_(*t*), *C*_*lv*_(*t*) and *C*_*rv*_(*t*) for actuating the *ra*, *la*, *lv*, and *rv* respectively. The compliance function across *ra* is given by Eqs (9) and (10), where *C*_*min*,*ra*_, *C*_*max*,*ra*_ are the minimum and maximum values of the *ra* compliance and *u*(*t*) is the activation function. The time *t* is considered over a complete cardiac cycle. *T*_*a*_ is the start of the activation of *ra* and *T* is the end of the cardiac cycle. The same is repeated for every cardiac cycle, where the temporal characteristics of the electrical activation are utilized from the EP model.

$$\begin{array}{cc} C_{ra}\left(t\right) & =C_{min,ra}+0.5\times \left(C_{max,ra}-C_{min,ra}\right)u\left(t\right) \end{array}$$ (9)

$$\begin{array}{cc} u(t) & =\{\begin{array}{cc} 0, & 0\leq t<T_{a}\\ 1-\cos(2\pi \frac{t-T_{a}}{T-T_{a}}), & T_{a}\leq t<T \end{array} \end{array}$$ (10)

Similarly, the compliance function across *la* is modeled using Eq (11), where *C*_*min*,*la*_, *C*_*max*,*la*_ are the minimum and maximum values of the *la* compliance, *u*(*t*) is the activation function of *la* as previously defined in Eq (9), and *d*_*la*_ represents the delay in activation of *la* with respect to *ra* (Fig 3a).

$$\begin{array}{c} C_{la}\left(t\right)=C_{min,la}+0.5\times \left(C_{max,la}-C_{min,la}\right)u\left(t-d_{la}\right) \end{array}$$ (11)

Likewise, the compliance functions across *lv* and *rv* can be represented as given by Eqs (12) and (13), where *C*_*i*_;*i* ∈ {*lv*, *rv*} is the systolic compliance across *lv* or *rv*, *u*_*v*_(*t*) is the activation function, and *d* represents the delay in activation of *lv* or *rv* from *ra*. *T*_1_ and *T*_2_ are the systolic and diastolic activation time instances of the cardiac cycle respectively.

$$\begin{array}{cc} C_{i}\left(t\right) & =C_{i}\times u_{v}\left(t-d\right),\;i\in \left\{lv,rv\right\} \end{array}$$ (12)

$$\begin{array}{cc} u_{v}\left(t\right) & =\{\begin{array}{cc} 0.5-0.5\cos(\pi \frac{t}{T_{1}}), & 0\leq t<T_{1}\\ 0.5+0.5\cos(\pi \frac{t-T_{1}}{T_{2}-T_{1}}), & T_{1}\leq t<T_{2}\\ 0, & T_{2}\leq t<T \end{array} \end{array}$$ (13)

Once, the above compliance functions are evaluated, they are directly applied to the Eqs (1)–(6), to compute the pressure dynamics in the cardiac chambers and systemic and pulmonary circulation. The delays (*d*_*la*_, *d*) and activation time instances (*T*_*a*_, *T*_1_, *T*_2_ and *T*) of the compliance functions for the cardiac cycle, are derived from electrocardiography (ECG) signal. The same is generated using a Forward EP pipeline, as discussed next.

### Electrophysiology model

The electrophysiology (EP) model mainly comprises for solving the forward problem, i.e, calculating body surface potential from a known heart potential [30]. This section uses the same procedure as described in one of our prior publication [31] to execute the Forward EP pipeline using ECGsim [32]. ECGsim is based on a biophysical model that connects the transmembrane action potential of representative myocytes on the heart surface to electrocardiogram (ECG) signal on the surface of the body. The cardiac source model is expressed as an equivalent double layer (EDL) of sources on the closed surface of the atria and ventricles and is analogous to an equivalent source of the currents generated at the cell membrane during depolarization of a myocyte, referred to as transmembrane potential (TMP). The cardiac surface is divided into a triangular mesh of 1500 elements or nodes, each such node poses an equivalent source, which is proportional to TMP of the nearest myocyte. Therefore, the Source matrix (*S*) at node *n* at the time *t* is defined as given in Eq (14) where, *D* is the depolarization phase, *R* is the repolarization phase. The timing of local depolarization at node ‘*n*’ is denoted as *δ*, timing of local repolarization at node ‘n’ is defined as *ρ* (Fig 1). The interval *α* = *ρ* − *δ* is taken as a measure of the local action potential duration. Such timing parameters and TMP amplitudes can be varied to induce different EP conditions.

$$\begin{array}{c} S(t;\delta ,\rho )=D(t;\delta )R(t;\rho ) \end{array}$$ (14)

Based on the EDL source description, local strength at position ‘*x*’ on the surface of the myocardium (*Sv*) can be mapped to potential *ϕ* generated at location ‘*y*’ on the body surface as given in Eq (15) where, *B*(*y*, *x*) is the transfer function expressing the volume conductor model, considering geometry and conductivity in the chest cavity, *V*_*m*_ is the local transmembrane potential at heart surface and *dω*(*y*, *x*) is the solid angle subtended at *y* by the surface element *dS*(*x*) of the myocardial node *Sv*.

$$\begin{array}{c} \phi \left(t,y\right)=\int B\left(y,x\right)V_{m}\left(t,x\right)d\omega \left(y,x\right) \end{array}$$ (15)

Volume conductor model as expressed in Eq (15) cannot be solved analytically due to the complex asymmetrical shape of individual compartments; rather, it is solved numerically, using a specialized Boundary element method (BEM) algorithm. The potential at discretized body surface consisting of ‘*ϕ*(*t*, *l*)’ lead position are expressed as given in Eq (16) where *B* is a transfer matrix, incorporating the solid angles subtended by source elements as viewed from the nodes of the triangulated surface.

$$\begin{array}{c} \phi \left(t,l\right)=\sum_{n}B(l,n)S(t;\delta ,\rho ) \end{array}$$ (16)

The resulting matrix ‘*ϕ*’ generates the body surface potential, a subset of which is the standard 12 lead ECG and single-lead ECG configuration. The generated single-lead ECG (Fig 3a) serves as the driving signal for the hemodynamics.

### Parameter estimation of compliance function from ECG signal

Coupling of EP and hemodynamics is done through a compliance function, which determines the time-varying compliance of atrium and ventricle and generates the systolic and diastolic functions of the heart. Single lead ECG is decomposed to its characteristic constituents like PQ (atrial depolarization), QRS (ventricular depolarization) and ST duration (ventricular repolarization), and R-R interval (Fig 3a). These timing information are used to generate a time-varying compliance function, which is used to control the synchronized operation of all the cardiac chambers.

Let us assume that $e\left(i\right)=\left[e_{1},e_{2},...,e_{n_{i}}\right]\in {ℜ}^{1\times n_{i}}$ represents the single lead ECG signal, for the *i*^*th*^ cardiac cycle. It comprises a time-series signal of *n*_*i*_ samples, having an amplitude of *e*_*j*_ for the *j*^*th*^ sample as shown in Fig 3a. To determine the activation parameters, as presented in Eqs (9)–(13), we need to perform the following operations

1. For determining the cardiac cycles, we first compute the R-R interval from the ECG signal. Let us assume that *D* = [*t*^1^, *t*^2^, …, *t*^*q*^] are the set of duration between the R-R peaks those are used to derive the cardiac cycles *e*(*i*).
2. For each cardiac cycle, *e*(*i*); *i* ∈ *q*, there exists a set of PQRST [33] time-instances represented by $[t_{P}^{i},t_{Q}^{i},t_{R}^{i},t_{S}^{i},t_{T}^{i}];i\in q$ as shown in Fig 3a.
3. For *i*^*th*^ cardiac cycle, the activation parameters are estimated as given in Eq 17 (Fig 3a)
  $$\begin{array}{c} T_{a}^{i}=t_{P}^{i};\;d_{la}^{i}=\frac{t_{P}^{i}+t_{Q}^{i}}{2};\;d^{i}=\left(t_{R}^{i}-t_{P}^{i}\right);\;T_{1}^{i}=\frac{t_{R}^{i}+t_{T}^{i}}{2};\;T_{2}^{i}=t_{T}^{i};\;T^{i}=t^{i} \end{array}$$ (17)
4. Therefore, for the entire ECG signal, comprising *q* cardiac cycles, we obtain a set of activation parameters $T_{act}=[\begin{array}{cccc} T_{act}^{1} & T_{act}^{2} & ... & T_{act}^{q} \end{array}]$, where, $T_{act}^{i}=[\begin{array}{cccccc} T_{a}^{i} & d_{la}^{i} & d^{i} & T_{1}^{i} & T_{2}^{i} & T^{i} \end{array}]$.
5. Based on each $T_{act}^{i};i\in q$ set, the activation signals (referred to Eqs (9)–(13)) are generated for all the cardiac chambers. One of such instance is shown in Fig 3b.

### Chamber and valve dynamics

Based on the valvular functions of the heart, there are four phases of operation during a cardiac cycle [34]. The dynamic equations realizing the cardiac phases, illustrate the interaction of pressure and volume of the cardiac cycle.

#### Cardiac phases

**Fig 4:**
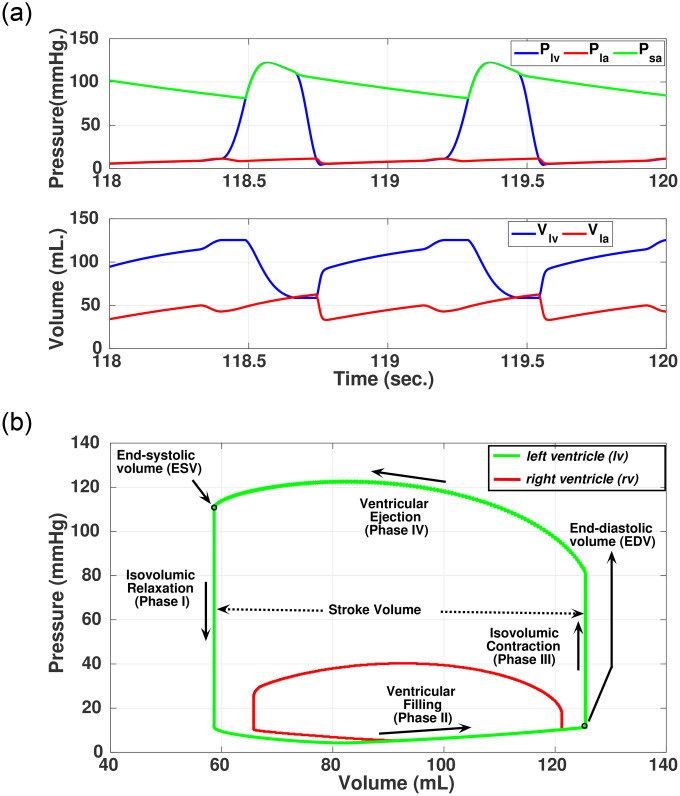
Simulated pressure, volume signals and loops in healthy cardiac condition. (a) Simulated pressure and volume waveforms across *lv*, and *la*. (b) Simulated PV-loops across *lv*, and *rv*.

Within each cardiac cycle *e*(*i*), having a duration of *T*^*i*^, four different phases of operation in *lv* can be observed based on the valvular functions of the left heart. The *lv-*pressure (*P*_*lv*_) and *lv-*volume (*V*_*lv*_) are used to generate pressure-volume (PV) loop [35] in each cardiac cycle as shown in Fig 4b. Using *Assumption* 1, the parameter *V*_*lv*_ can be evaluated as given by Eq (18).

$$\begin{array}{c} {\dot{V}}_{lv}=C_{lv}\left(t\right){\dot{P}}_{lv}+{\dot{C}}_{lv}\left(t\right)P_{lv} \end{array}$$ (18)

The four cardiac phases are briefly mentioned here.

#### Isovolumetric relaxation (Phase I)

In this phase of operation, both the valves in the left-heart are closed as the pressure in successor region is greater than the predecessor region; hence, *U*_*mi*_ = *δ*_*mi*_ = 0 and *U*_*ao*_ = *δ*_*ao*_ = 0. In this phase, the pressure in the *lv* decreases, however, the volume remain constant as all the valves are closed. The volume of blood that remains in *lv* is called as the end-systolic volume (ESV). The *la* pressure continues to rise because of the venous return from the pulmonary system.

#### Ventricular filing (Phase II)

After the relaxation phase, at some point, the pressure in *la* would be greater than pressure in *lv*, thus, *P*_*la*_ > *P*_*lv*_. As a consequence, the mitral valve will open (*U*_*mi*_ = 1) that subsequently allows the blood to flow from *la* to *lv*. Hence, the *lv* volume increases. However, in this phase, the pressure in *sa* will be greater than the pressure in *lv*, so, the aortic valve will remain closed, therefore, *U*_*ao*_ = *δ*_*ao*_ = 0.

#### Isovolumetric contraction (Phase III)

After the filling of *lv*, the mitral valve closes because of the increased intra-ventricular pressure, thus *U*_*mi*_ = *δ*_*mi*_ = 0. During this period, the aortic valve still remains closed, thus *U*_*ao*_ = *δ*_*ao*_ = 0. In this phase, the *lv* pressure starts increasing, without changing the *lv* volume, as all the valves are closed. The volume of blood that remains in *lv* is called as the end-diastolic volume (EDV).

#### Ventricular ejection (Phase IV)

After the isovolumic contraction phase, the intra-ventricular pressure starts increasing. When it exceeds the *sa* pressure (i.e. *P*_*lv*_ > *P*_*sa*_), the aortic valve opens (*U*_*ao*_ = 1). As a consequence, the blood from *lv* is ejected via aorta. Hence, the *lv* volume decreases.

The above mentioned four phases occur in a cyclic manner and primarily drives the overall hemodynamics. A representative variation in pressure and volume in *la*, *lv* and *sa* are shown in Fig 4a. Concise information on the working of the cardiac phases is best represented by the Pressure-Volume (PV) loops (Fig 4b). From the PV-loop of the left ventricle, physiological parameters such as stroke volume (SV), cardiac output (CO), ejection fraction (EF), end-diastolic (EDV) and end-systolic (ESV) volume can be estimated [35]. These parameters provide information on the inadequate filling of the *lv* during diastole or insufficient ejection in a systolic phase or whether the cardiac function is normal. The interaction of the pressure-volume of the right ventricle during the ventricular phases is also shown in Fig 4b.

#### Estimation of cardiac chamber diameters

Considering the shape of each cardiac chamber to be ellipsoidal, hence, the diameter of a cardiac chamber can be calculated from its respective volume as given in Eq 19 [29], where *k*_*i*_ is a scale factor, and *l*_*i*_ is the length of the *i*^*th*^ chamber. In this current work, we have only estimated the diameters for the *la*, and *lv*, that can provide indications of possible enlargement or hypertrophy in those chambers.

$$\begin{array}{c} d_{i}=\sqrt{\frac{6V_{i}}{4\pi k_{i}l_{i}}},\;i\in \left\{la,lv\right\} \end{array}$$ (19)

### Modeling of Photoplethysmogram (PPG) signal

Photoplethysmogram signal captures the temporal volumetric change in blood flow by means of detecting the reflection or absorption of incident light on the capillaries of the systemic circulation [36]. Usually this is taken from the fingertip or ear lobes using a combined photo transmitter and detector type of device. The PPG signal is affected by the hemodynamics of the heart, physiology of the peripheral vessels and the heart rate. A typical PPG signal represents two separate flows, the *lv* contraction causing the intra-arterial pulse pressure wave in the proximal aorta and the reverse flow during filling of the *rv*. These two flows give rise to a large systolic peak and a small diastolic peak as shown in Fig 6c. Additionally, on the falling edge of PPG, a dichroic notch is also observed just before the diastolic peak. It is to be noted that the amplitude of the PPG signal is unit-less. Hence the relative temporal relationship between the peaks and notches along with the morphology of the signal, are used to extract the combined pathophysiological condition of the heart and systemic circulation. The steepness of the rising slope of the systolic peak in PPG, is found to be correlated with the blood pressure [37]. Likewise, features derived from the derivatives of the PPG signal, can provide indications of change in arterial stiffness [23], or indications of atherosclerosis. In such cases, the blood flow is effected due to the intimal thickening of the arteries and blood vessels.

In one of our recent works, such effects in the PPG are simulated in the systemic circulation [38]. In the proposed PPG function [38], we have modeled the similar consequences while employing the *sa*-pressure dynamics (*P*_*sa*_) for the forward flow, and the *ra*-pressure dynamics (*P*_*ra*_) for the reverse flow. Hence, the function of the modeled PPG signal (*p*(*t*)) is represented as given in Eq (20).

$$\begin{array}{c} p\left(t\right)=\frac{k_{1}\times P_{sa}\left(t\right)-k_{2}\times P_{ra}\left(t\right)}{R_{s}} \end{array}$$ (20)

where *k*_1_ and *k*_2_ are the forward and reverse coefficients to replicate the arteries and arterioles of the systemic circulation. These coefficients are initially tuned to match the experimentally measured PPG signals for healthy subjects. Such a combined cardiovascular and PPG model is then used to study the changes in the peripheral flow, where the hemodynamics is effected due to disorders in mitral valve.

## Simulating mitral valve disorders

Mitral valve (MI) dysfunctions are a major medical problem worldwide [39]. MI disorders include ‘stenosis’ and ‘regurgitation’ of valve. In the former case, the blood flow through mitral valve experiences additional obstruction during diastolic *lv* filling phase. For the later, during systole, due to improper functioning of the valve, a portion of the stroke volume passes retrogradely from *lv* to *la*. The next two sections focus on the dynamics associated with MI disorder.

### Simulation of mitral stenosis

In mitral stenosis (MS), the orifice of the valvular area gets narrower. As a consequence, a high resistance across the stenotic valve causes blood to stay inside the *la*, thus raising the *la*-pressure (*P*_*la*_). Hence, the *la*-volume (*V*_*la*_) increases (enlargement) over time because it has to produce higher pressure when it contracts against the high resistive stenotic valve. Moreover, the mechanical obstruction leads to an increase in pressure within the pulmonary vasculature and the right side of the heart. The reduced filling in *lv* decreases the ventricular stroke volume (SV), thus the cardiac output (CO) and the aortic pressure (*P*_*sa*_) also reduce. MS is mostly caused by rheumatic heart disease or thickening, shortening, fusion, and calcification of the chordae tendineae [40]. Progression of MS eventually leads to the development of disabling symptoms like dyspnea, thromboembolism, and right-sided heart failure.

Normally, the mitral valve has an effective area of 4–6 cm^2^ [40]. During MS, this area gets decreased. Based on this reduction in area, MS have been graded [41] as mild, severe, and very severe as shown in Table 1. However, pathological symptoms are usually observed once the stenosis is in the severe or very severe range [41].

**Table 1:** Severity of MS based on the valvular area [41] (normal ≥4 *cm*^2^).

Parameter	Mild	Severe	Very Severe
Valve area (*cm*^2^)	>1.5	1.5–1	<1
% reduction of valve area compared to normal	<62.5%	62.5%–80%	>80%

During MS, the blood flow through MI is reduced because of the increasing valvular resistance. In our study, we have created similar scenarios by varying the parameter *R*_*mi*_ of Eqs (1) and (2). Therefore, depending on the geometric orifice area of the stenotic mitral valve (i.e. severity of MS), the diseased $R_{mi}^{d}$ is calculated as shown in (21)

$$\begin{array}{c} R_{mi}^{d}=\frac{A_{MS}}{A_{N}}\times R_{mi} \end{array}$$ (21)

where, *A*_*N*_ = 4 *cm*^2^, *A*_*MS*_ is the geometric orifice area of the stenotic mitral valve, and *R*_*mi*_ = 0.002 mmHg.sec/mL defines the resistance across MI in normal condition [29]. So, based on the severity of MS (Table 1), the estimated $R_{mi}^{d}$ are presented in Table 2. Because of these changes of mitral resistances during MS, the ventricular-filling phase is affected which will subsequently shift the isovolumic contraction and ventricular ejection phases by reducing the *lv*-pressure (*P*_*lv*_) and *lv*-end-diastolic volume (LVEDV).

**Table 2:** Simulation of MS severity (*R*_*mi*_ = 0.002 mmHg.sec/mL).

Parameter	Mild	Severe	Very Severe
$R_{mi}^{d}$ (mmHg.sec/mL)	0.01	0.03	0.1
% of simulated stenosis	55%	70%	86%

### Simulation of mitral regurgitation

MI regurgitation or insufficiency causes blood to leak backward through the mitral valve when the heart contracts [42]. This reduces the amount of blood pumped out to the body. The literature suggests that a trivial amount of mitral regurgitation (MR) is present in up to 70 percent of adults [43]. Significant MR is mostly due to the abnormality in a heart valve like mitral valve prolapse or other cardiac diseases like endocarditis, rheumatic fever, etc. MR can develop as a result of other types of heart diseases, such as after a heart attack or other cause of heart muscle injury [43]. As it is associated with *lv* and atrial volume overload, at the advanced stages, it forces cardiac remodeling, effecting chamber dilation with clinical consequences of atrial fibrillation, and heart failure [43].

In the study, the effect of the backward blood flow, due to the improper closing of the mitral valve at the ventricular ejection phase, is modeled by the parameter *δ*_*mi*_ in Eqs (1) and (2), under the assumption that *δ*_*mi*_ > 0. Therefore, in isovolumic contraction and ventricular ejection phases, some amount of blood flows in the backward direction (from *lv* to *la*) based on the value of *δ*_*mi*_.

Hence, *δ*_*mi*_ defines the regurgitation severity, indicating the dysfunction of the parameter *U*_*mi*_ during *P*_*la*_ < *P*_*lv*_ as given in Eq (7) under *otherwise* condition. During MR, there is a volume increment in the *la* and *lv*. As a result, enlargement and hypertrophy evolve across those chambers to support the larger stroke volume (SV). The level of severity of MR is usually measured by regurgitation fraction (RF), which is defined as the percentage of *lv* stroke volume that regurgitates into *la* as given in Eq 22, where *V*_*mi*_ and *V*_*ao*_ are the forward blood volume of the mitral and aortic valve respectively during a cardiac cycle.

$$\begin{array}{c} RF=\frac{V_{mi}-V_{ao}}{V_{mi}}\times 100\% \end{array}$$ (22)

Hence, based on the parameter *δ*_*mi*_, the simulated RF can be evaluated from the forward and backward blood flow through the mitral valve. Mild, moderate, and severe MR have been graded [44] as displayed in Table 3.

**Table 3:** Severity of MR based on regurgitation fraction (RF) [44].

Parameter	Mild	Moderate	Severe
RF	<30%	30%–49%	>49%
Simulated RF	23%	45%	89%
*δ*_*mi*_	0.004	0.024	0.05

## Results

Simulations have been executed on a system having 8 GB of RAM with *Intel core-i7* processor in MATLAB software environment. The following parametric values [25], as shown in Table 4, are used to perform the simulation.

**Table 4:** Constant parameters employed in CVS model during simulations.

Parameters	Value	Physiological Significance	Parameters	Value	Physiological Significance
*Resistances (mmHg. sec/mL)*					
*R*_*p*_	0.01	Pulmonary	*R*_*s*_	0.05	Systemic
*R*_*ao*_	0.002	Aortic valve	*R*_*tr*_	0.001	Tricuspid valve
*R*_*pu*_	0.001	Pulmonic valve			
*Compliances (mmHg. sec^2^/mL)*					
*C*_*sa*_	0.2	Systemic	*C*_*pa*_	5	Pulmonic
*C*_*min*,*ra*_	0.2	*ra* minimum	*C*_*max*,*ra*_	0.3	*ra* maximum
*C*_*min*,*la*_	0.2	*la* minimum	*C*_*max*,*la*_	0.3	*la* maximum
*C*_*lv*_	2.5	*lv* end systolic	*C*_*rv*_	1	*rv* end systolic
*Unstressed Volume (mL.)*					
*V*_*lv*,*s*_	15	*lv*	*V*_*la*,*s*_	5	*la*
*V*_*ra*,*s*_	5	*ra*	*V*_*rv*,*s*_	40	*rv*
*Constant coefficients and length of chambers (cm.)*					
*k*_1_	1	Systolic PPG	*k*_2_	2.5	Diastolic PPG
*l*_*la*_	5.5	length of *la* chamber	*k*_*lv*_	1.15	*lv* scaling factor
*k*_*la*_	1.2	*la* scaling factor	*l*_*lv*_	8	length of *lv* chamber
*Initial conditions (mmHg.)*					
$p_{la}^{0}$	5	left atrium (*la*)	$p_{lv}^{0}$	5	left ventricle (*lv*)
$p_{sa}^{0}$	80	systemic arteries (*sa*)	$p_{ra}^{0}$	5	right atrium (*ra*)
$p_{rv}^{0}$	5	right ventricle (*rv*)	$p_{pa}^{0}$	15	pulmonary artery (*pa*)

### Simulation of normal cardiac condition

To study the performance of the proposed hemodynamic cardiovascular system (CVS) for a normal cardiac condition, representing the cardiac dynamics of a healthy adult, we set the mitral valve resistance (*R*_*mi*_) to 0.002 (shown in Table 2) and *δ*_*i*_ = 0, *i* ∈ {*mi*, *ao*, *tr*, *pu*}. From the Table 5, it is seen that most of the physiological parameters such as stroke volume (SV), cardiac output (CO), ejection fraction (EF), mean arterial pressure (MAP), etc. are within the physiological range, obtained by actual measurements on health subjects, as suggested in literature [45]. Moreover, the *lv* end-diastolic (LVEDD), end-systolic (LVESD) dimensions and *la* end-diastolic (LAEDD) dimension are also within the range experimental observations [45].

**Table 5:** Evaluated cardiac parameters. As experimentally reported for healthy subjects [29, 45] versus the simulation output of the proposed approach.

Parameters	Experimental Value	Simulated Value
LVEDD (in cm)	3.8–5.2	4.9
LVESD (in cm)	2.3–3.9	3.48
LAEDD (in cm)	3.8	3.7
LVEDV (in mL)	125	124.9–125.5
LVESV (in mL)	55	55.05–58.64
Stroke Volume (in mL)	70	69.85–66.86
Heart Rate	75	75
Cardiac Output (L/min)	5.2	5.2–5.01
Ejection Fraction (%)	56	55.92–53.3
Mean Arterial Pressure (in mmHg)	70–100	101

**Fig 5:**
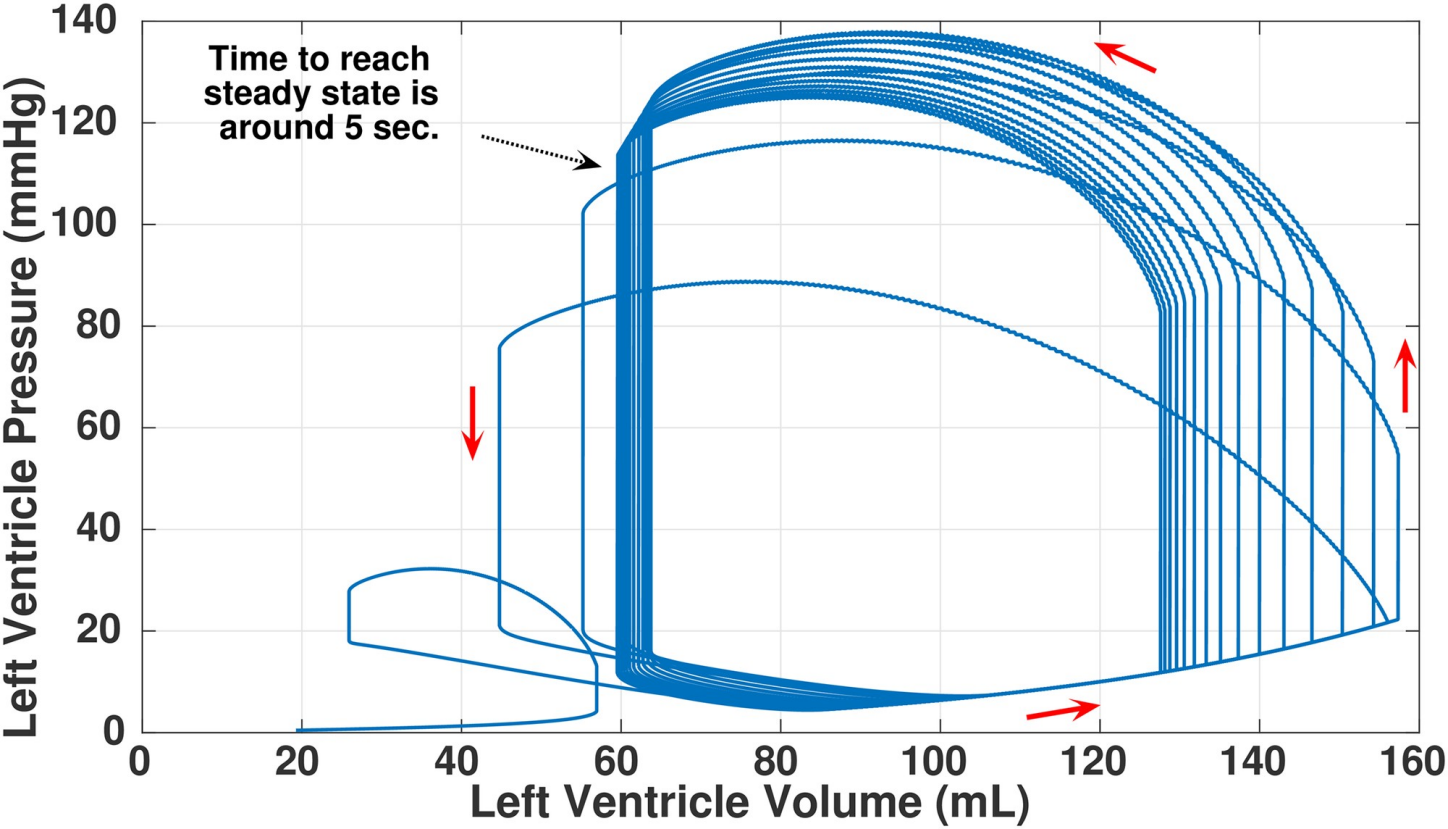
Stabilization of limit cycle for PV-loops of *lv*. For reaching to a continuous limit-cycle in the PV state-space, the simulation takes 5 sec of time.

The simulations are performed by solving the differential equations as stated in Eqs (1)–(6). For a given cardiac condition, it takes approximately, 5 sec to reach the steady-state value and 0.2 sec to complete one loop (using *Intel core-i7* processor with 8 GB of RAM). In case of a normal cardiac condition, an example of such initial time progression of PV-loop of *lv*, before it reaches the steady state, is shown in Fig 5. We can see the trajectory in which the pressure and volume of *lv* changes with time and finally forms a continuous limit-cycle in the PV state-space.

### Mitral stenosis

**Fig 6:**
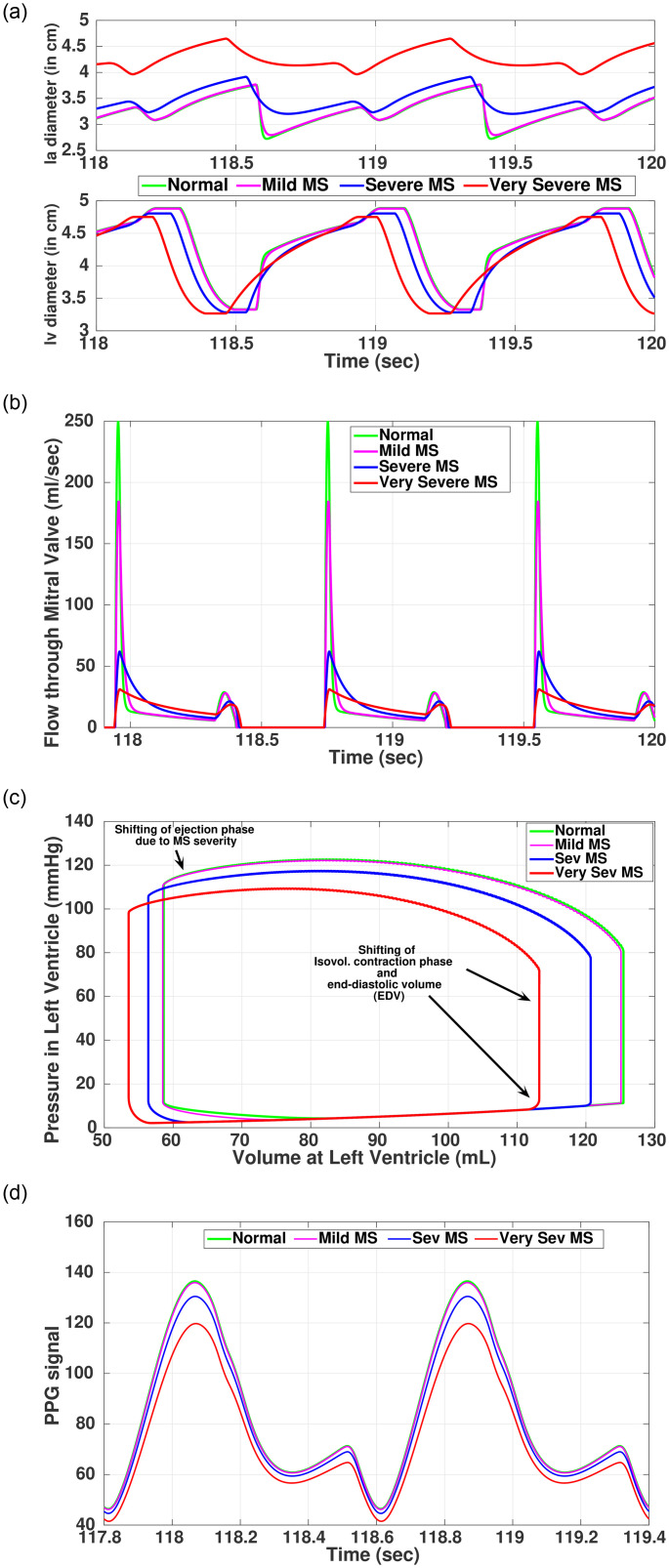
Simulated outcomes of Mitral stenosis with progression of disease. (a) Valvular diameters in MS, (b) Blood flow through Mitral valve, (c) PV-loop left ventricle, (d) Simulated PPG signal.

In mitral stenosis, as severity increases with reduction in orifice area, high resistance across the mitral valve causes blood to accumulate in the left atrium (*la*), increasing *la* pressure and subsequent increase in *la* volume, compensating for decreasing *lv* volume. As the *la* increases in size, there is an increase in pulmonary pressure, often causing pulmonary congestion and increased risk of developing atrial fibrillation. The severity of ‘stenosis’ has been modeled by the parameter *R*_*mi*_ (Table 2). Based on the severity, Fig 6 shows progressive changes in *la* and *lv* chambers diameter (Fig 6a), flow through mitral valve (Fig 6b), *lv* PV-loop (Fig 6c), and PPG signal (Fig 6d). As anticipated, flow through the valve decreases considerably as the disease progress.

From the *lv* PV-loop (Fig 6c), it is evident that with increasing severity of MS, the ventricular filling phase is mostly affected, which subsequently shifts the isovolumic contraction and ventricular ejection phases while reducing *P*_*lv*_ and LVEDV. In light of the above consequences, several physiological parameters are estimated from the *lv* PV-loop as shown in Table 6. Parameters like CO, SV, EF, MAP represent a decreasing trend with increasing severity of stenosis. Two important parameters, naming End-Systolic-Pressure-Volume-Ratio (ESPVR) (representing the ionotropic state of the ventricles) and End-Diastolic-Pressure-Volume-Ratio (EDPVR) (reciprocal to the ventricular compliance) have also been calculated. With progression of MS from severe to very severe, myocardial contractility is shown to be depressed, as indicated by a decreasing trend in the ESPVR index. Similarly for EDPVR, a decreasing trend as the disease progresses suggests a possible ventricular remodeling, where compliance increases due to dilated heart chambers [46]. The most common indicator of MS is the *la* enlargement. This feature is faithfully reproduced in our simulation, where it is seen that with increasing severity, the left atrial end-diastolic diameter (LAEDD) has been enlarged with respect to the normal value thus signifying the enlargement condition in *la*.

**Table 6:** Simulated parameters in MS.

Parameters	Simulated value for mild MS	Simulated value severe MS	Simulated value very severe MS
LVEDD (in cm)	4.9	4.81	4.75
LVESD (in cm)	3.475	3.43	3.34
LAEDD (in cm)	3.75	3.9	4.55
ESPVR	1.89	1.88	1.84
EDPVR	0.24	0.21	0.1884
LVEDV (in mL)	125.1	120.7	113.2
LVESV (in mL)	59.04	56.39	53.54
Stroke Volume (in mL)	66.06	64.31	58.67
Cardiac Output (L/min)	4.95	4.82	4.4
Ejection Fraction (%)	55.5	53.1	50.5
Mean Arterial Pressure (in mmHg)	101	98	91

Simulated PPG signal relates systemic blood pressure and change in volumetric flow at the aorta to a distal measurement site like a fingertip. The simulation result for MS is shown in Fig 6d. With the increasing severity, the relative amplitudes of the systolic peaks are reduced because of less volume in *lv* and decreased MAP [46]. However, the diastolic peaks or the dichroic notches are nearly identical for all the cases.

### Mitral regurgitation

MR pathology and evolution of clinical symptoms are well documented in the literature and are subdivided into three dominant phases that correlate with disease progression from mild to severe [43]. The first phase is termed as ‘Compensated phase’ representing mild to moderate disease progression where the major change is an enlargement of *lv*. During this phase, there is no physical manifestation of symptoms. The next phase is termed as the ‘Transitional phase’ where the weakening of myocardium starts and ventricles can no longer compensate for the leak due to regurgitation. Clinical symptoms like fatigue (decreased ability to exercise), shortness of breath begin to appear in this stage. As the disease progresses further, it enters into the ‘Decompensated phase’. In this phase, there is a progressive enlargement of the *la* and abnormal heart rhythm, mostly Atrial Fibrillation (AF) manifests. Blood pressure in pulmonary arteries increases giving rise to pulmonary hypertension. These changes slowly lead to heart failure like condition.

**Fig 7:**
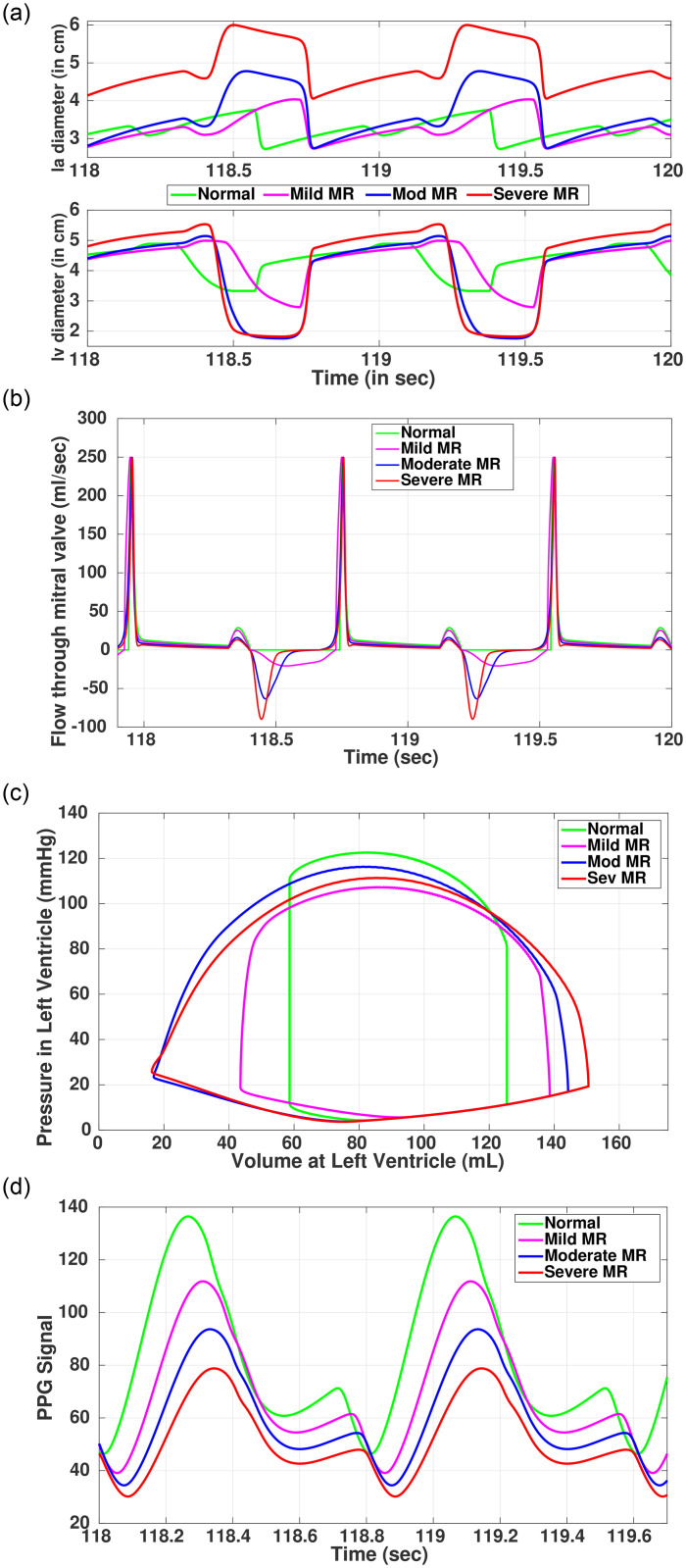
Simulated outcomes of Mitral regurgitation. (a) Valvular diameter, (b) Blood flow through Mitral valve, (c) Left ventricle PV-loop, (d) Simulated PPG signal.

In our simulation results (as shown in Fig 7 and Table 7), the effects of the above-mentioned phases are reflected as the disease progresses. The results for *lv* dynamics (Table 7) closely matches with the medical reports [45]. Moreover, while comparing the diameters, the hypertrophy and enlargement are seen for the ventricular and atrial chambers respectively. As per the medical literature, these phenomena are usually observed for MR patients. As discussed previously, in MR, a fraction of backward blood flow through MI, during the ventricular ejection phase. This backflow rises with increasing severity. Subsequently, there is a marked increase in the volume of *la* as shown in Fig 7a. With increasing severity, the pressure across *la* is enhanced, thus, the velocity of the blood flow during the ventricular filing state and the ejection phase increase. Additionally, a reverse flow of blood from *lv* to *la* occurs giving rise to the mitral flow profile, as shown in Fig 7b as the negative values. PV loop of *lv* (Fig 7c) shows characteristics typical to MR, with no prominent isovolumic relaxation or contraction phases. As the width of the PV-loop raises, the total stroke volume increases but the forward flow reduces. The increased ventricular total SV includes the volume of blood ejected into the aorta as well as the volume ejected back into the *la*. Thus, even if the ejection fraction of MR subject shows above 55%, which is normal for a healthy case, the forward SV is substantially reduced for severe MR.

**Table 7:** Simulated parameters in MR.

Parameters	Simulated value for mild MR	Simulated value for moderate MR	Simulated value for severe MR
LVEDD (in cm)	4.75	5.15	5.5
LVESD (in cm)	2.9	1.9	1.85
LAEDD (in cm)	4	4.5	6
ESPVR	1.74	2.07	2.36
EDPVR	0.285	0.33	0.36
LVEDV (in mL)	138.6	144.4	150.5
LVESV (in mL)	63.30	59.50	60
Forward Stroke Volume (in mL)	68.7	66.8	59.7
Total Stroke Volume (in mL)	75.27	84.90	90.5
Cardiac Output (L/min)	5.15	5.01	4.48
Ejection Fraction (%)	54.30	54.15	54.13
Mean Arterial Pressure (in mmHg)	88	86.75	80

As MR progresses, afterload on the ventricle is greatly reduced, reducing end-systolic volume (Table 7). Ventricular end-diastolic volume and pressure increase due to elevated pressure in *la* and also due to ventricular remodeling to keep the ventricular compliance in check. As the disease progresses, the slope of EDPVR increases, reflecting the effect of ventricular hypertrophy and the heart muscle becoming stiff or less compliant. As a compensatory mechanism to maintain cardiac output and arterial pressure, ventricles adapt by increasing the size, contractility, and heart rate. This often gives rise to arrhythmia [45]. Increased contractility, as the disease progresses, is reflected by the increase in the ESPVR index (Table 7).

In MR, as the disease progresses, there is a reduction in MAP coupled with cardiac output due to the effect of regurgitation, ventricular hypertrophy, and altered ionotropy. Pulmonary hypertension further reduces oxygenated blood circulation in the systemic loop. Due to the reduced forward flow and low oxygen concentration, the peaks at systolic and diastolic peaks in PPG signal shows marked reduction in relative amplitude (Fig 7d) as the disease progresses.

### Hemodynamic response to recorded ECG signals

To validate the applicability of the proposed model, we have employed three real ECG data from the Physionet PTBI dataset [47] taken from patients with mitral-valve disorders. From the medical history, as provided in the dataset, it is perceived that the patients with IDs 107, 110, and 188 are suffering from severe MS, severe MR and moderate to severe MR with AR respectively. The hypertrophies/enlargements in the cardiac chambers have a strong relationship with the ECG signal. There are existing literature [33, 48, 49] describing one-to-one relationship between the cardiac hypertrophy/enlargement and the ECG signal. In this paper, we have utilized the Romhilt-Estes point-score system [50] to quantify the enlargement and hypertrophy in *la* and *lv* respectively. The score for *la* enlargement is generated using the ECG-criteria measured from Lead II and V1 [51–53]. The ECG-criteria for *lv* hypertrophy is dependent on multiple factors ranging from voltage criteria to ST-segment abnormality [54, 55]. A score of less than 15 and 5, indicate a normal *la* and *lv* respectively. Higher the scores, more are the effects of hypertrophy, with a maximum possible score of 13 for *lv*, whereas there is no such upper bound for *la*. For benefit of the readers, the methodology to derive the scores, is briefly presented in Appendix. Additionally, the detailed time and voltage information obtained from the ECG data of the above mentioned three patients (IDs 107, 110, and 188) are shown in the Table 9. These information are then used to derive the hypertrophy/enlargement scores using the ECG-criteria and given in the last two rows of Table 8.

**Table 8:** Simulated hemodynamic parameters with measured ECG.

Parameters	Patient ID 107	Patient ID 110	Patient ID 188
Heart rate (bpm)	91.35±1.48	73.77±0.44	85.66±0.42
LVEDD (cm)	4.63±0.3	5.05±0.5	5.77±0.5
LVESD (cm)	3.3±0.26	2.05±0.41	1.97±0.44
LAEDD (cm)	4.75±0.1	5.72±0.2	5.16±0.32
ESPVR	1.8±0.2	2.11±0.37	2.04±0.09
EDPVR	0.19±0.2	0.32±0.36	0.3±0.1
LVEDV (mL)	90.25±0.3	125.32±0.5	93.62±0.5
LVESV (mL)	42.42±0.26	50.91±0.41	35.3±0.44
Forward Stroke Volume (mL)	47.83±0.72	71.43±0.48	49.15±0.4
Cardiac Output (L/min)	4.37±1.45	5.27±0.5	4.21±0.56
Ejection Fraction (%)	53.0±2.1	56.93±0.4	52.5±0.77
Mean Arterial Pressure(mmHg)	92±5.3	75±2.5	52±4.1
Enlargement score (la), (normal <15)	20	20	20
Hypertrophy score (lv), (normal <5)	4	11	11

**Table 9:** Temporal and amplitude related information obtained from the ECG signals of three patients from Physionet PTBI dataset [47], having mitral valve disorders.

Patient Id		107	110	188
Valve disease		*Severe MS*	*Severe MR*	*Mod to Sev MR with AR*
**Lead II**	*Duration between two peaks of P wave (millisec.)*	45	101	78
	*Total P wave duration (millisec.)*	121	155	147
**Lead V1**	*Duration of biphasic P wave with terminal negative portion (millisec.)*	67	76	70
	*Amplitude of biphasic P wave with terminal negative portion (millimeter)*	≥1	1.25	≥1
**Lead I**	*Amplitude of R wave (millimeter)*	5	12	17.5
**Lead III**	*Amplitude of S wave (millimeter)*	5	19	10.5
**Lead V1**	*Amplitude of S wave (millimeter)*	5	35	37
**Lead V2**	*Amplitude of S wave (millimeter)*	4.8	32	34
**Lead V5**	*Amplitude of R wave (millimeter)*	5	25	45
**Lead V6**	*Amplitude of R wave (millimeter)*	5	29	42
*ST-T vector opposite to QRS without digitalis*		*no*	*yes*	*yes*
*ST-T vector opposite to QRS with digitalis*		*yes*	*no*	*no*
*Left atrial enlargement*		*yes*	*yes*	*yes*
*QRS duration (millisec.)*		75	115	125
**Lead V5**	*Duration of delayed intrinsicoid deflection (millisec.)*	35	55	≥50
**Lead V6**	*Duration of delayed intrinsicoid deflection (millisec.)*	42	49	≥50

Next, in order to the drive the CVS model, we extract the required temporal parameters (using Eq 17) from the respective ECG-signal (from lead-V2) of the above three patients. In this aspect, we would like to mention that as the gradation of valvular disease (i.e. mitral valve area) is not mentioned in the dataset, we have run the model by changing the valvular resistances as per the severity ranges. The simulated hemodynamic parameters from the CVS model, for all the three patients, are tabulated in Table 8.

The enlargement score obtained from the ECG-data of patient ID 107, indicates high possibility of the presence of *la*-enlargement, whereas, it is low for *lv*-hypertrophy. Similarly, patient IDs 110 and 188, the possibilities of presence *la* enlargement and *lv* hypertrophy are high. Executing the CVS model with the ECG signal, for the above-mentioned patients, we have observed similar consequences. Simulated parameters corresponding to left ventricular end-diastolic dimension (LVEDD) and left atrial end-diastolic dimension (LAEDD) (marked using red box in Table 8), correspond with the hypertrophy and enlargement scores calculated using Romhilt-Estes point-score system respectively.

From the meta-data of patient IDs 110 and 188 (shown in Table 9), it is seen that both of the patients have severe MR. In addition to that, patient ID 188 has concurrent Aortic Regurgitation (AR) along with MR. As per the medical report [56], it is witnessed that the patient with concurrent MR and AR, is having a large end-diastolic left-ventricular diameter (LVEDD) than the patient with only MR. Simulating the model with the respective ECGs of the above patients, it is seen that LVEDD of ID-188 is greater than the patient with ID-110. The results are shown in Table 8.

Comparing the *lv*-end-systolic-diameters (LVESD) of the patient IDs 188 (having concurrent AR with moderate to severe MR) and 110 (severe MR), it is perceived that the LVESD for both cases is approximately within a similar range (Table 8). The main reason behind this fact is that here, MR is more effective than AR. If we consider the ejection fraction for both the cases, we would observe that the patient having concurrent AR with MR is having less ejection fraction than the patient having only MR because the contractile dysfunction across *lv* is leading to reduce the *lv* ejection fraction. Therefore, from the above analysis, we can conclude that our proposed solution can handle the polyvalvular disease.

## Discussion

Simulation results for the mitral valve disorders reflect that the developed model can capture the cardiac dynamics, thus creating an efficient platform to study combinations of physiological and pathophysiological cardiac and vascular properties as well as the effects of potential modifications in the system dynamics. Compared with the existing reduced-order hemodynamic models [26, 29, 45], our proposed model provides a detailed four-chambered heart model with EP coupling, capable of simulating mitral valve disorders in real-time execution (5 seconds to reach steady-state condition). Another advantage of our proposed model over the existing hemodynamic model [19] is the PPG generation capability.

Although there is no direct clinical evidence of PPG signal morphology changing with MS or MR progression, synthetic PPG actually may help in deciphering classification problem for early detection of disease. PPG signal can be analyzed in several ways, both morphologically and statistically. Features from such signals are useful in designing a machine learning-based classifier for disease prediction [38, 57–59].

An important application of the proposed model is the capability to create different ‘what if’ analysis based on the changes in the physical, pathological, or ambulatory condition of the subject. To demonstrate one such use case, we have created a ‘what if’ analysis of stress or exercise condition on subjects with mitral stenosis and regurgitation valve disorders. This ‘induced stress’ behavior is an important diagnostics criteria for valve disorder patients [60].

**Fig 8:**
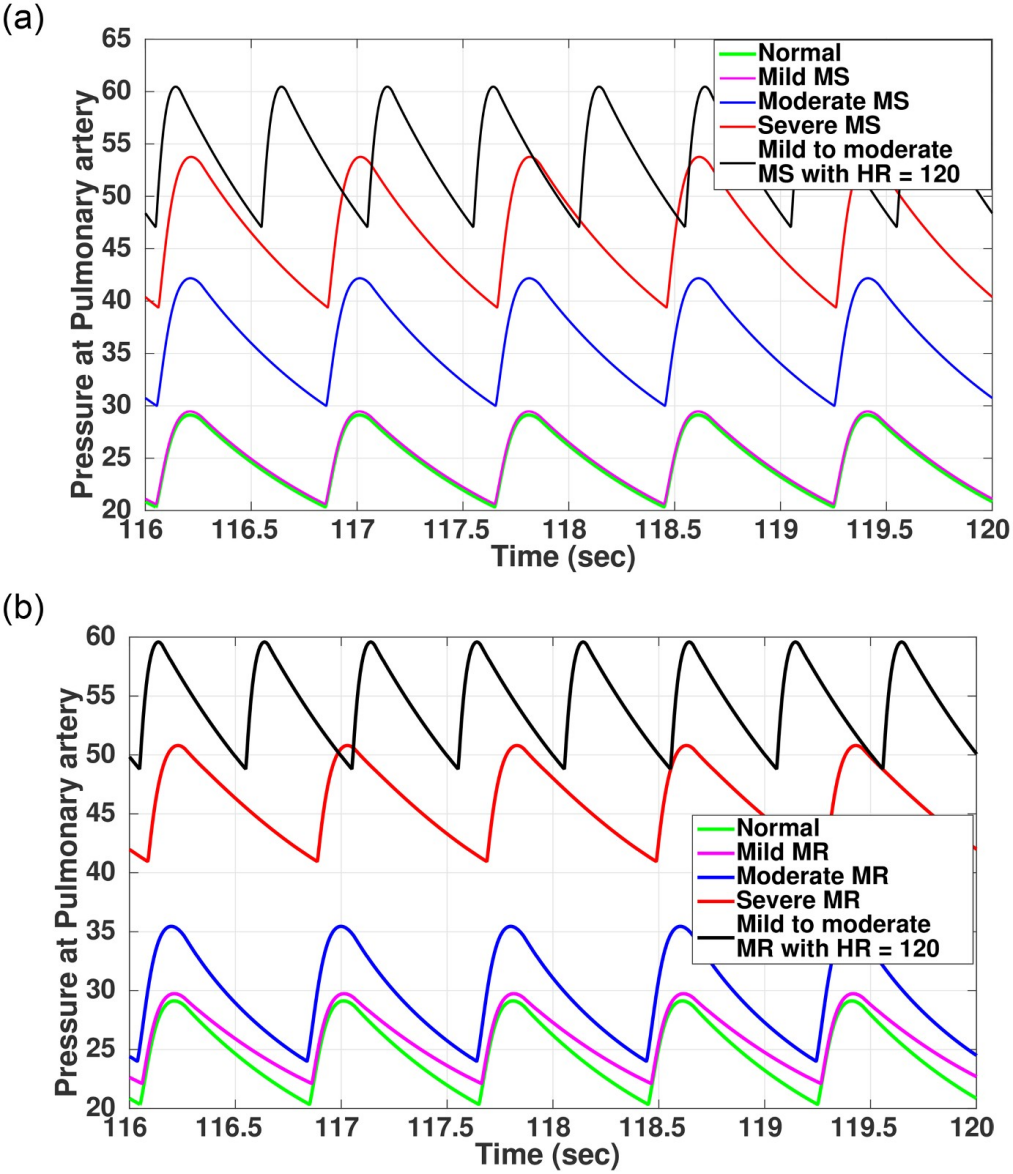
Induced stress behavior on pulmonary pressure (mmHg) for MS and MR. (a) Pulmonary pressure (MS), (b) Pulmonary pressure (MR).

Pathophysiological symptoms associated with MS only get manifested in the moderate to severe region. Similarly, most people with mitral regurgitation (MR) have no symptoms till they are at the near severe stage, and abnormal heart rhythm starts generating due to ventricular remodeling or there is a sign of pulmonary hypertension [60]. However, stress or exercise condition may trigger MS and MR symptomatic behavior like shortness of breath, congestion in the chest, fatigue, and palpitation [27]. To simulate such an effect, we increased the heart rate to 120 beats/min to replicate the stress or exercise condition. A new ECG template was generated with modification in the source model to generate an ECG signal with an increased heart rate of 120 bpm. The compliance curve for the new ECG template was modified and fed to the hemodynamic module. Fig 8 shows the change in pulmonary pressure in MS and MR subject with mild disease progression under the altered condition as compared to the resting condition with heart rate of 75 bpm.

For MS, as indicated in Fig 8a, there is a momentary increase in pulmonary pressure (*P*_*pa*_). This sudden increase in *P*_*pa*_ due to stress, coupled with varying changes in physiology due to onset of stenosis, gives rise to symptoms for diagnosis which under normal resting condition is not manifested until the disease progresses to a significant level [27]. Similarly, for MR (Fig 8b) there is a temporary increase in *P*_*pa*_, increasing the workload of the right side of the heart, making it difficult to provide an adequate amount of oxygenated blood to the body and manifesting symptoms of breathlessness, pulmonary congestion, and fatigue [27].

The holistic approach of integrating the cardiac source model and electrophysiology further provide enhancements to the hemodynamics that can be used to aid clinical decision support tools. With the proposed coupled electrophysiology-hemodynamics system, we demonstrate a pipeline for ECG synthesis from cardiac TMP, which in turn triggers and modulates the hemodynamic chain of operations. However, the proposed model can also be fed-in with measured true ECG and generate the hemodynamic parameters. This feature enables to estimate hemodynamic features, clinically revealed through echocardiography study to be predicted by the model only with ECG information. The ECG genesis mode is more suitable for EP driven diseases like myocardial ischemia [31] or conduction disorders like arrhythmia, where cardiac TMP gets modified due to the underlying disease condition. For valvular diseases, TMP effects are observed in a later period due to cardiac remodeling and a true mechano-electric feedback model needs to be incorporated to bring in the effect of ventricular hypertrophy or atrial enlargement in the ionic process of electrical conduction in heart tissue. Lack of this mechano-electric feedback path is a limitation of our current model. The proposed model, due to lack of actual sufficient valvular disease data may not provide quantitative justification, however, a trend can be validated based on the pathophysiological reports and understandings [61]. Another limitation of this model is to capture the changes in ventricular wall dimension due to remodeling effect, noticed after prolonged disease condition or with condition like ischemia or bundle brunch disorders.

The concept of cardiac remodeling and associated ventricular shape changes due to change in sarcomere contraction and calcium dynamics is a topic of active research and we are working on integrating electrophysiology and cardiac biomechanics to replicate some of the aspects of cardiac remodeling. We are also working on the effect of ischemia and its effect on hemodynamic parameter variation [31]. Presently, the effect of regional wall motion abnormality is out of scope for the cardiac model. However, we are working on developing the mechanical wall motion changes with disease progression and hope to integrate the concept in our existing model in near future.

Further improvement in the model in the line of integrating biomechanical properties to the time-varying compliance model, will further enhance the effect of ‘preload’ and ‘afterload’ phases and will also increase the plethora of ‘what if’ analysis. EP model can be further enhanced to introduce the effect of arrhythmia, as seen in advanced stages of the valvular disease, and also incorporate the effect of ventricular remodeling on the sarcomere excitation-contraction mechanism and its eventual effect on the hemodynamics.

## Conclusion

In this paper, we report the development of a multiscale-multimodal cardiac model to capture the changes in hemodynamics due to mitral valve disorders. The proposed approach couples electrophysiology with hemodynamics, enhancing a holistic understanding of underlying pathophysiological changes associated with mitral valve stenosis and regurgitation. The simulated results of the healthy condition and diseased conditions are in accordance with clinical reports. The developed model and the simulation platform can establish the trend of physiological parameters with disease progression. Real-time simulation of the multiscale model may aid in the personalized cardiac model generation that can be used to understand the qualitative interaction of cardiac variables and also aid in patient-specific outcome prediction.

The uniqueness of the developed model is not any particular advanced modeling solutions in each sub-part, but rather the integrated holistic approach giving a comprehensive overview of cardiovascular hemodynamics in healthy and mitral valve disease in an in-silico environment. The possibility to change the model parameters and create ‘what if’ analysis facilitates a comprehensive understanding of pathological manifestation, whereas the real-time simulation and feedback impart features that could be used in a clinical decision support scenario in intensive care or surgical use cases. The model will be further enhanced to improve the understanding of complex hemodynamic states and validation through patient data.

## Appendix

### A Computation of Scores for la Enlargement and *lv* Hypertrophy from the ECG Signal

The criteria to generate the score for la enlargement from the Lead II and V1 of ECG signals are briefly given below [52].

- For ECG lead II, the duration between two peaks of the bifid P wave, that is greater than 40 milliseconds, and the net duration of the P wave, that is greater than 110 milliseconds.
- For lead V1, the duration of the terminal negative portion of the biphasic P wave, that is greater than 40 milliseconds, and the amplitude of the terminal negative portion of the biphasic P wave, that is greater than 1 millimeter (0.1 millivolt).

For the assessment of the *lv* hypertrophy, the scores are computed from the time and amplitude of various segments in ECG signals [55]. These criteria are briefly given below.

- Voltage criteria—(i) The amplitude of R wave or S wave in limb leads is greater or equal to 20 millimeters, (ii) the amplitude of S wave in Leads V1 or V2 is greater or equal to 30 millimeters and (iii) the amplitude of R wave in V5 or V6 is greater or equal to 30 millimeters. If any of these three criteria is satisfied then the score would 3.
- Abnormality in ST segment—(i) If the ST-T vector is opposite to QRS without digitalis then the score is 3, (ii) if the ST-T vector is opposite to QRS with digitalis then the score is 3.
- Additional conditions—(i) If the left-atrial enlargement is present then the score is 3, (ii) if QRS duration greater than or equal to 90 milliseconds, then the score is 1, (iii) delayed intrinsicoid deflection in Leads V5 or V6 is greater than 50 milliseconds, then the score is 1.
- Finally, adding all the above scores, if it is greater than 5, then the possibility of the presence of *lv*-hypertrophy is approximately 83%-97% [50].

## Supporting information

S1 Data (ZIP)
